# Characteristics, Diagnosis and Treatment of Compound Odontoma Associated with Impacted Teeth

**DOI:** 10.3390/children9101509

**Published:** 2022-10-02

**Authors:** Marta Mazur, Gianni Di Giorgio, Artnora Ndokaj, Maciej Jedliński, Denise Corridore, Beatrice Marasca, Alessandro Salucci, Antonella Polimeni, Livia Ottolenghi, Maurizio Bossù, Fabrizio Guerra

**Affiliations:** 1 Department of Oral and Maxillofacial Sciences, Sapienza University of Rome, 00161 Rome, Italy; 2 Department of Interdisciplinary Dentistry, Pomeranian Medical University in Szczecin, 70-111 Szczecin, Poland

**Keywords:** compound odontoma, odontoma, transmigration, impaction, developmental age, oral surgery

## Abstract

Compound odontoma is a malformation typical of young adults below the age of 20, with a slight preference for the male gender and the anterior region of the maxilla. Clinically asymptomatic, it can be detected during a radiological investigation in connection with the persistence of deciduous dental elements and the impaction of definitive ones. The treatment of choice is excisional surgery and recurrence is a rare event. The need for orthodontic therapy for impacted elements is usually not necessary because in most cases, odontomas are small, circumscribed lesions the size of a permanent tooth. In this article, the diagnostic and therapeutic surgical excision procedure is presented in three patients at developmental age with large compound odontomas associated with at least one retained canine, and in two of the cases, with serious transmigration to the impacted tooth elements.

## 1. Introduction

Odontomas are hamartomatous developmental malformations of the dental tissues [1]. According to the World Health Organization (WHO), a compound odontoma is defined as “a malformation in which all dental tissues are represented in a more orderly pattern than in the complex odontoma, so that the lesion contains many tooth-like structures. Most of these structures do not morphologically resemble the teeth in the normal dentition; however, enamel, dentin, cementum and pulp are arranged as in the normal tooth” [2,3].

Odontomas are developmental anomalies consequential to the growth of totally differentiated epithelial and mesenchymal cells that generate ameloblasts and odontoblasts [4]. These cells and tissues can look either normal or not fully developed in structure. The level of differentiation in the formed tissues can be variable, and both enamel, dentin, cementum, and pulp may be present within the compound odontoma [3]. The complex odontomas are characterized by non-descript masses of dental tissues, while compound odontomas by multiple, well-formed tooth-like structures [3].

Typically asymptomatic, they are revealed on routine radiographs or upon assessing the origin of delayed tooth eruption [1,5,6,7,8]. Radiographically, depending on the development stage, they may appear as radiolucent in the initial phase and as a radiopaque form at progressive stages [5]. The diagnosis is based on clinical examination and radiographic images, and following surgical removal, it must be further confirmed by histological examination. Differential diagnosis is made with all other ossified bone lesions, such as ossifying fibroma, odontoameloblastoma, ameloblastic fibroma or fibro odontoma, osteoma and fibrous dysplasia; or florid osseous dysplasia [5] to decide the most appropriate treatment [9]. 

Very rarely, the spontaneous eruption can occur with exposition within the oral cavity of the odontoma, and it may be associated with pain, localized inflammation, or infection with suppuration [6].

Odontomas can be diagnosed at any age and in any location of the oral cavity, but more frequently during the second decade of life, on average at 14.8 years [3]. Odontomas show an incidence of 22–67%, being the most common odontogenic tumours [6]. Males (59%) and the anterior maxilla (67%) are more frequently affected by odontomas [3,9].

Odontomas are commonly asymptomatic and constitute casual findings. Clinical signs may be delayed eruption and persistence of the deciduous teeth. In severe cases, infection or regional lymphadenopathy may be observed [9].

Management usually consists of surgery to prevent further complications with the permanent tooth eruption in the paediatric population [4,10], and the prognosis after treatment is favourable, with scant relapse [9].

Following surgical removal of the odontoma, orthodontic therapy is often not prescribed to reposition any impacted tooth elements [11]. In the great majority of cases, the removed odontoma is smaller than a tooth by size, and the impacted tooth is not highly deviated; if the root is still forming, it will manifest full potential to erupt. Radiological assessment of the developmental status of the impacted tooth root can be helpful in deciding which therapeutic choice to make on a case-by-case basis [11,12].

This article presents three clinical cases of patients at developmental age diagnosed with large compound odontomas, associated with the inclusion of at least one permanent canine and impacted teeth transmigration in two of the cases. The diagnostic process and surgical treatment of choice are described, in which the two transmigration cases led not only to the removal of the odontoma but also to the extraction of the impacted and transmigrated tooth elements. In all cases, histopathological diagnosis was performed and was compatible with compound odontoma.

Besides, literature searches of free text and MeSH terms were performed using PubMed and Google Scholar from 2000 to 2022. All searches were conducted using a combination of subject headings and free-text terms. The keywords used in the search strategy were as follows:

(“compound odontoma” AND “complex odontoma”) AND (“paediatric age” AND “children” OR “developmental age”) AND (“tooth impaction” OR “impacted teeth” OR “impacted tooth” AND “tooth transmigration”).

The aim of this review was to hightlight the anatomical, demographic, gender and dental parameters associated with complex and compound odontoma.

Inclusion criteria were as follows: (a) prospective studies; (b) retrospective studies; (c) case series; (d) in-vivo studies; (e) studies published in English, French, Polish and Albanian. These criteria have been broadly selected to be as sensitive as possible. The exclusion criteria were as follows: (a) in-vitro studies; (b) articles without statistical analysis; (c) abstracts and letters to the editor.

A manual search was also conducted to try to find other additional studies as well. Based on the inclusion criteria, two authors (MM and GDG) reviewed the titles and abstracts and selected the studies from the literature independently. The full text of each study was then read to decide whether it could be included or not. Disagreements in this case were set on by consensus between both authors or by discussion with another author (AN).

### 1.1. Case Number 1

**Figure 1:**
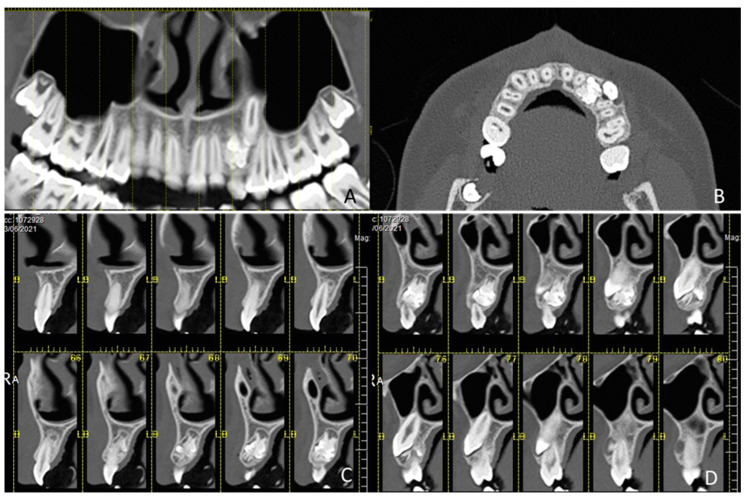
Series of images from cone beam computed tomography (CBCT). (**A**) Orthopanoramic CBCT view shows the persistence in the arch of the upper left deciduous canine 63 and the overlapping presence of a compound odontoma, which in its evolution blocked the eruption of the corresponding permanent canine; (**B**) A cross-section image highlights the permanent canine that has been displaced vestibular due to the presence of an odontoma that is palatal to the canine; (**C**,**D**) Parasagittal images that show that the odontoma completely occupies the entire thickness of the bone between the buccal and palatal cortex. Absence of bone trabeculae and displacement of the permanent canine towards the vestibulum.

**Figure 2:**
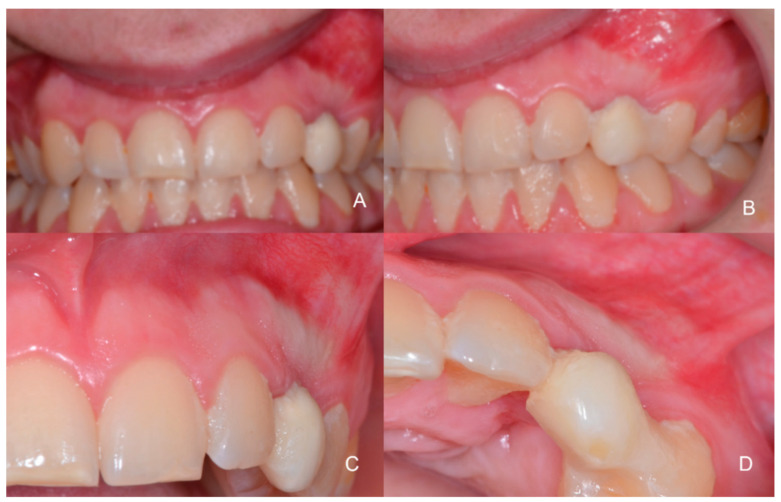
Intra-oral photographic images at baseline. (**A**,**B**) Front and side view before surgery; (**C**,**D**) Detail in the lateral and occlusal view with a temporary prosthesis in place 23. An expansion of the bone cortex is visible in line with the odontoma and the permanent area of the canine.

**Figure 3:**
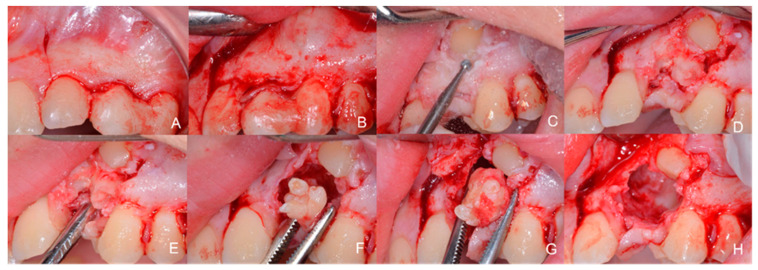
Intraoperative images. (**A**) L-shaped flap with a convex indentation on element 22 and an intrasulcular incision extending to the tooth 24; (**B**,**C**) Removal of the temporary denture, exposure of the canine with a ball bone cutter (bone cutter ball head 018 Meisenger, Neuss, Germany); (**D**) Exposure of the canine; (**E**) Onset of exposure of the odontoma; (**F**) Enucleation of odontoma, good cutting plane visible; (**G**) Detail of the odontoma structures that are removed together; (**H**) Extensive residual bone fissure palatal to the position of the canine.

**Figure 4:**
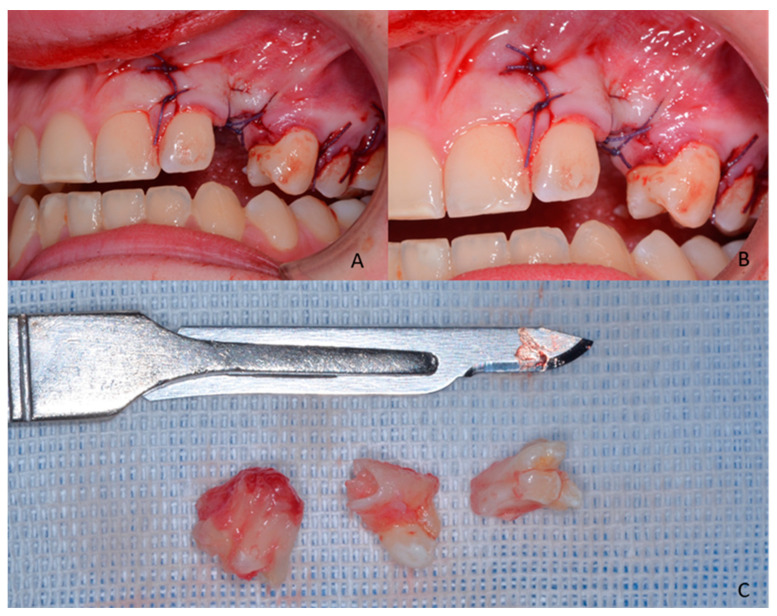
(**A**,**B**) Suture of the flap (Vicryl Ethicon 3.0, 17 mm 1/2c, Johnson & Johnson International, Hamburg, Germany); (**C**) clinical aspect of the odontoma, (Bard-Parker stainless steel size 15, Benefits srl, Genova, Italy). (**A**,**B**) The suture is visible at the level of the relief incision in the ridge; (**C**) various fragments of the removed odontoma are recognizable, the neoformation is organized in variously cusped denticles. The structures of the compound odontoma with crowns and roots are recognizable.

A 14-year-old female patient, treated in our paediatric surgery unit in 2021 (Figure 1, Figure 2, Figure 3 and Figure 4).

### 1.2. Case Number 2

**Figure 5:**
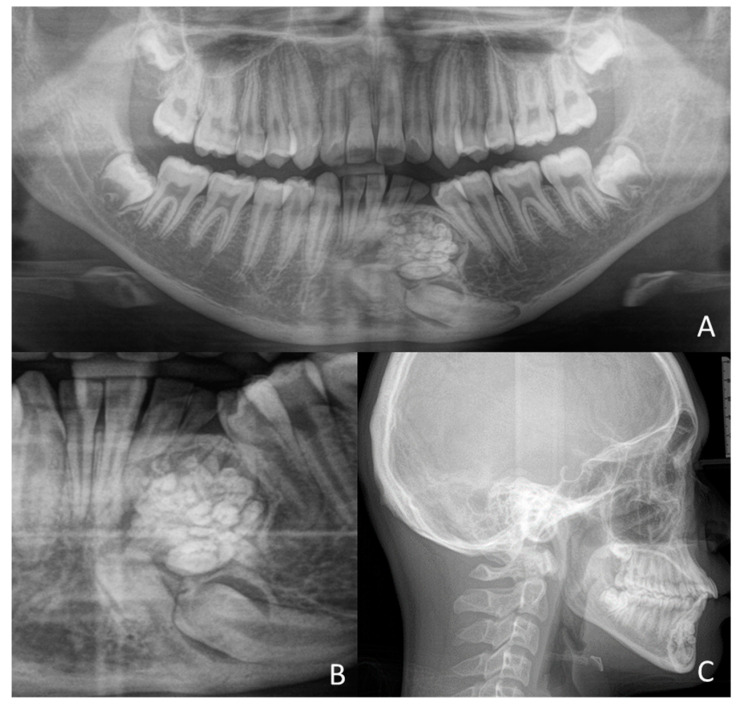
(**A**) Orthopanoramic radiography with the presence of a compound odontoma, formed by many teeth, recognizable on the radiographic image. The maintenance of the deciduous element 72 and the inclusion of the left permanent canine 33 at the lower edge of the mandible. Moreover, mesial to the location of element 33, the presence of element 32 is marked; (**B**) Detail of compound odontoma, the presence of all the teeth forming the new formation; (**C**) Cranial teleradiograph in lateral–lateral projection showing the neoformation, the width of the symphysis is completely occupied by the odontoma.

**Figure 6:**
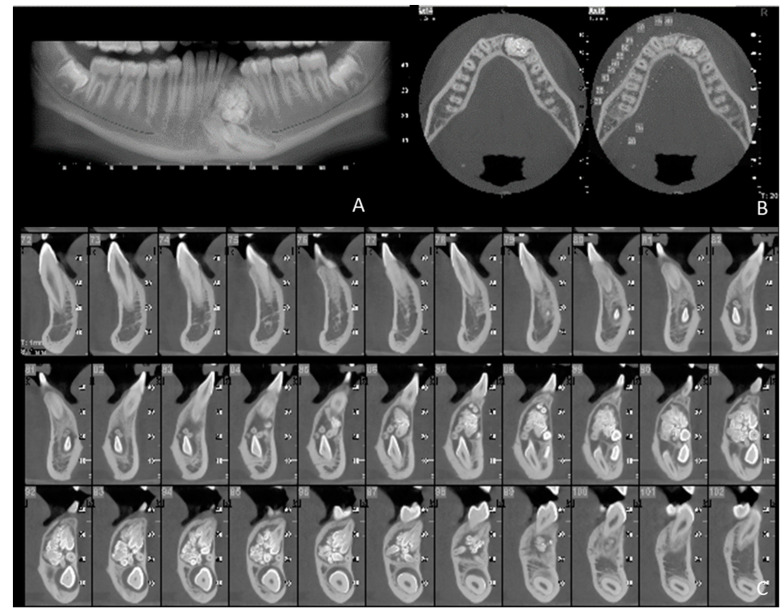
Series of images from cone beam computed tomography (CBCT). (**A**) The localization of the neoformation is anterior to the emergence of the mandibular nerve, which is at the level of element 34. During the surgical phase, the emergence of the nerve is highlighted in order to ensure its preservation. It is again possible to highlight the denticles; they are arranged with various degrees of angulation. The whole odontoma is positioned superiorly with respect to the permanent canine, which is in the inferior portion of the mandible; (**B**) The presence of the neoformation in the bone structure of the mandible. The vestibular cortex is thinned, absent in some places; (**C**) Axial slices showing lesion-thinning cortical plates and the compound odontoma occupying the entire sagittal thickness of the mandible in the upper part. Visible are the tooth-like structures, some fused together, others not fused, with different sizes. Canine 33 and lateral incisor 32 have been moved towards the mandibular caudal cortex in a more horizontal position, certainly raised by canine 33, presenting with hyperplastic dental follicle.

**Figure 7:**
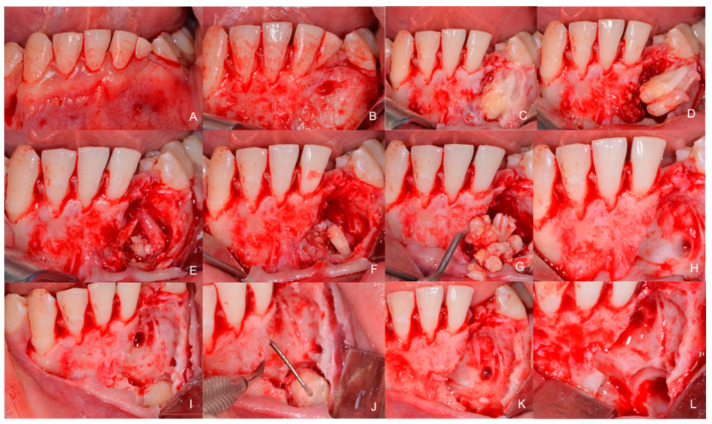
Intraoperative images. (**A**) “L”-shaped flap with the first incision with a distal discharge cut at 43 and then the other intrasulcular incisor along all the lower incisors, including the deciduous one, up to the level of 34; (**B**) Complete skeletonization of the bone. A veil of cortical bone above the odontoma is present; (**C**) Removal of the bone covering the odontoma and the various denticles are extracted (**D**–**G**), on the images the denticules appear with a cluster or a cauliflower organization; (**F**) A slight capsule outside the odontoma is present; there is a good cleavage plane with respect to the underlying bone and there are no adhesions; (**H**) Complete removal of the compound odontoma; (**I**) The lower portion of the included canine begins to be seen. The crown of the canine is freed using the bone burs (bone cutter ball head 018 Hager & Meisinger GmbH, Neuss, Germany) on a straight handpiece (KaVo TYP Surgical straight handpiece dental, Genova, Italy), then the root (**J**,**K**) is also cut and extracted, as otherwise it would have been extremely difficult to extract the whole tooth; (**L**) Extension of the area and of the large bone defect following the surgical removal of the odontoma and the included dental elements.

**Figure 8:**
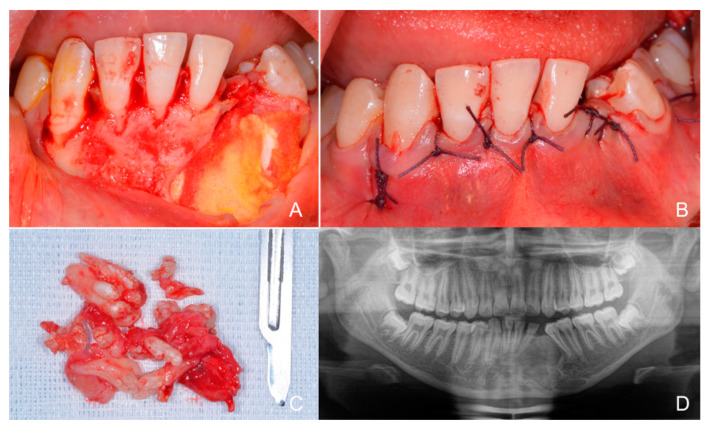
Post-surgery images. (**A**) The bone defect is filled with a fibrin sponge (Spongostan Dental, absorbable haemostatic gelatine sponge, Ethicon, Somerville, MA, USA), which guarantees a good clot; (**B**) The suture (Vicryl Ethicon 3.0, 17 mm 1/2c, Johnson & Johnson International, Hamburg, Germany) that recomposes the nature of the tissues is visible; (**C**) The odontoma clinical findings consisting of an ensemble of calcified structures, some like mini-teeth, some denticles appear as single-rooted, others as multi-rooted, some even fused, with no complete root formation and enamel, dentin, and cement being identified as dental tissues. (**D**) The orthopanoramic image after 6 months showing the progressive reconstruction of the bone anatomy of the area. The odontoma resulted in both dislocation and subsequent inclusion of 32 and 33, but also caused root displacement of 31, 41, and 34. Any type of orthodontic therapy is postponed; not only is bone formation required at all the entire area resulting from the surgical removal of the odontoma, but it is also necessary to check the vitality of the dental elements adjacent to the area itself.

The second clinical case concerns a 14-year-old male patient who was referred for consultation to our paediatric oral surgery unit. The patient was operated on in November 2021 (Figure 5, Figure 6, Figure 7 and Figure 8). The diagnosis of odontoma was made in conjunction with routine radiographic control, motivated by the lack of teeth 32 and 33 in the dental arch.

### 1.3. Case Number 3

**Figure 9:**
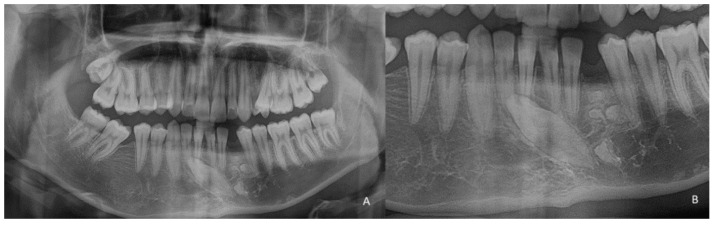
(**A**) Orthopanoramic image of the dental arches. The inclusion of the lower left canine, element 33, and the presence of at least four denticles are noted. The organization of a new formation is a little different than in the previous cases; (**B**) Details of the pre-operative orthopanoramic on the left. This seems less organized as can be seen in detail in (**B**); the various denticles seem less circumscribed and more scattered in the bone structure. The presence of the neoformation determined the inclusion of the tooth; there are no agenesis.

**Figure 10:**
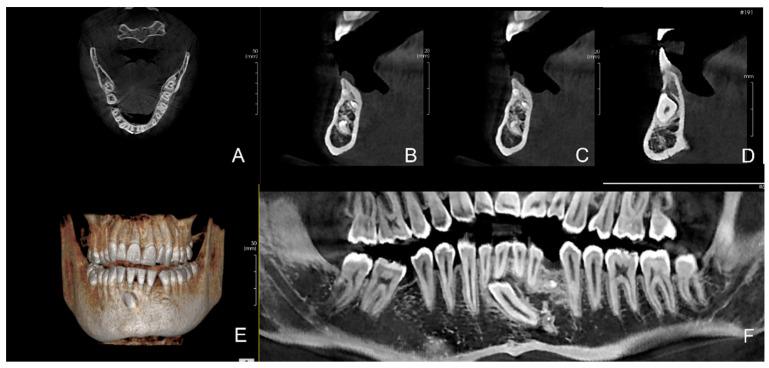
Series of images from cone beam computed tomography (CBCT) prior to the surgery. (**A**) The crown of the impacted canine 33 deforms the vestibular profile of the mandible; (**B**,**C**) Parasagittal images of the odontoma; (**D**) Parasagittal images, the canine can be seen in the vestibular position; (**E**) Three-dimensional reconstruction confirms the position of the canine which is at the level of the right central incisor 41; (**F**) Detail of the orthopanoramic image, showing the position of the canine in close contact with the root apexes of the central incisors, 41 and 31, and also partially of the lateral ones, 32 and 42.

**Figure 11:**
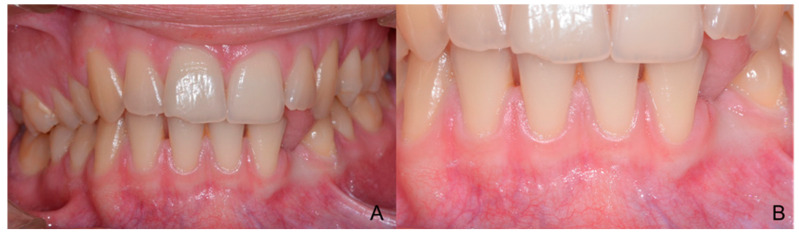
Intraoral photographic documentation before surgery. (**A**) Frontal view; (**B**) Particular of the frontal view in correspondence of the impacted canine upper area.

**Figure 12:**
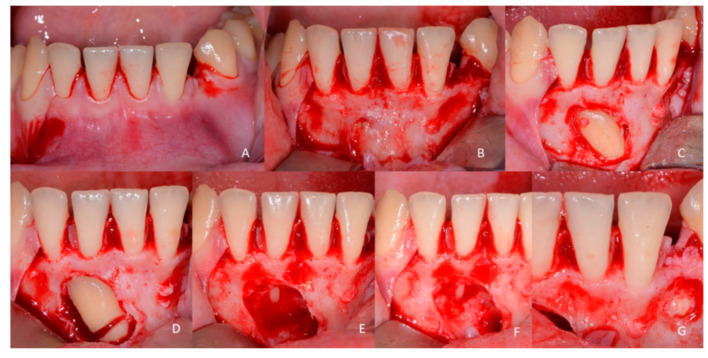
Pictures of the surgical procedure. (**A**) Flap design performed with the relief cut distal to 43, then within the gingival sulcus of the incisors; (**B**) After full thickness buccal flap the skeletonization of the bone, the included tooth is already visible, the bone is removed to remove the fibrotic sac present around the crown; (**C**) The crown is fully exposed; (**D**–**E**) To make it possible to extract the impacted canine, the tooth is cut, then the crown is extracted first and then the root; (**F**) After completing the extraction, the residual bone cavity and dental structure, if visible, is analysed. In this case, the root apex of 41 is visible and the vitality of this tooth will be evaluated during the follow-up; (**G**) The denticles of the odontoma begin to be exposed. In this case, there are only 4 single neoformations positioned in the bone structure and detached from each other, thus being even more difficult to find because they were not all four fused together.

**Figure 13:**
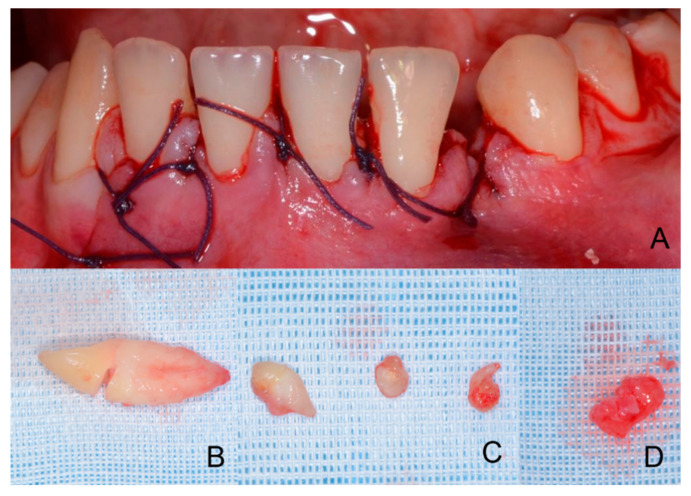
Post-operative images. (**A**) Frontal intraoral image with the suture of the flap (Vicryl Ethicon 3.0, 22 mm 1/2c, Johnson & Johnson International, Hamburg, Germany); (**B**) Image of the extracted canine; (**C**,**D**) The four extracted denticles which were part of the odontoma.

The third case presented here is that of a 15-year-old male patient. The surgery was performed in July 2021 (Figure 9, Figure 10, Figure 11, Figure 12 and Figure 13).

## 2. Literature Review

The search strategy identified 124 potential articles: 33 from PubMed and 91 from Google Scholar. After removal of duplicates, 91 articles were analysed. Subsequently, 71 papers were excluded because they did not meet the inclusion criteria and were not relevant to the subject of the study. The remaining 20 papers were included [13,14,15,16,17,18,19,20,21,22,23,24,25,26,27,28,29,30,31,32]. 

The studies included (Table 1) in the present review of the literature were published between 2002 and 2015. The total sample size of the analysed odontomas was 1279 (range: 11–163). All cases were given the subclassifications of complex or compound odontoma, and the ratio was 1:0.92. No gender predilection was seen in the overall sample, where the male to female ratio was 1:0.98. Information on the management of the involved teeth could not be obtained, as there were not available reports.

## 3. Discussion

Compound odontoma is a benign odontogenic tumour and it is usually diagnosed in young adults during regular radiological examination performed to assess the reason of a missing or mispositioned tooth in permanent dentition [33].

At the clinical level, compound odontoma can often be associated with anterior teeth misalignment and tooth eruption disorders, with possible impaction and delayed tooth eruption. One quarter of patients are asymptomatic, but compound odontoma can also be characterized by pain (13.3%) and swelling (8.9%) [34]. The preferred localization of compound odontomas is the anterior maxilla (81.8%) [33].

Surgical removal is the usual treatment and recurrence is rare [35]. 

In the clinical cases presented here, it was possible to refer the patient for orthodontic treatment of the impacted canine only in the first case, when the inclusion concerned the upper left canine. In this case, the odontoma blocked the physiological eruption of the canine, but did not cause it to be misaligned. In fact, the canine position was vertical, even if slightly vestibular, placed above the odontoma.

In the remaining two cases, the position of the odontoma was in the front part of the mandible and was associated with the inclusion of the canine, which was unfavourable for orthodontic treatment. In fact, in both cases, we proceeded with both the surgical enucleation of the odontoma and the extraction of the impacted canine, and in one case, also the extraction of the lateral impacted transmigrated incisor. In both cases, the large volume occupied by the odontoma, and late diagnosis certainly contributed to the displacement of the canine from its physiological site towards the mesial position, almost completely horizontal towards the bottom of the mandible on the inferior cortex, in line with the entire symphysis area of the chin. 

This type of tooth displacement is called transmigration. Transmigration most commonly concerns the permanent canines and is characterized by the movement of a non-erupted tooth that crosses the median line of the mandible and goes to position itself in the opposite part of the anterior mandible, as in cases 2 and 3 of this report [36]. Transmigration is rare, with an incidence of 0.007–0.08% and a 1.3:1 female predilection [37]. Interestingly, in this report, both cases of compound odontoma associated with transmigration of the left lower canine and lateral incisor were observed in male patients at developmental age. 

The presence of cysts and tumours can alter the eruption pathway and cause tooth transmigration. The transmigrated teeth typically present unilaterally with a certain degree of angulation. In case 2, the angulation was almost horizontal to the lower jawline, while the transmigrated canine in case 3 presented a 60-degree angulation to the corresponding long axis of the first lower premolar [38,39].

The treatment of choice for transmigrated canines in association with vast compound odontoma is extraction. Orthodontic reposition of transmigrated canines is usually reserved for mild cases [40].

Few cases in the literature could be found with transmigrated canines being transplanted following endodontic therapy [41].

However, the management of bone gaps after surgical excision treatment of large lesions may require regenerative approaches using a combination of three-dimensional structures and new therapeutic means [42].

## 4. Conclusions

This article has documented three cases of compound odontomas: one in the upper premaxilla and two in the mandible in patients at developmental age. All the cases were associated with canine impaction; however, only the maxillary-impacted canine underwent orthodontic treatment after surgery. The two mandibular canines associated with compound odontoma were transmigrated and no orthodontic therapy was possible due to the challenging near-horizontal position at the lower portion of the anterior mandibula.

Although the radiological and macroscopic features following surgical removal are pathognomonic, histological examination is always performed. As a diagnostic investigation in preparation for surgery, CBCT offers details on the morphology of the odontoma, its relationship with the surrounding dental structures, and the cortical bone profiles of the affected jaws.

## Figures and Tables

**Table 1:** Review of the literature related to compound and complex odontoma.

Author	Year	Analysed Odontomas	Compound	Complex	Female	Male
Boffano [13]	2012	52	20	32	26	26
Iatrou [14]	2010	26	15	9	12	14
An [15]	2012	73	45	28	38	35
El-Gehani [16]	2009	29	19	10	19	10
Ochsenius [17]	2002	163	71	92	85	77
Sekerci [18]	2015	35	24	11	24	11
Luo [19]	2009	80	33	47	38	42
da Silva [20]	2009	48	34	14	22	26
Taghavi [21]	2013	27	17	10	8	19
Hisatomi [22]	2002	106	62	44	51	55
Tomizawa [23]	2005	38	31	7	15	23
Amado [24]	2003	61	38	23	29	32
Pippi [25]	2006	28	15	13	11	17
Fernandes [26]	2005	85	33	52	38	47
Saghravanian [27]	2010	44	17	27	16	18
Jing [28]	2007	78	20	58	40	38
Soluk [29]	2012	160	57	99	80	80
Tamme [30]	2004	26	12	14	18	8
Olgac [31]	2006	109	42	67	50	59
Tawfik [32]	2010	11	7	4	8	3
Total		1279	612	661	628	640

## Data Availability

Not applicable.
